# Development of an Air Temperature Observation System Using a Radiation Shield and Neural Network Correction

**DOI:** 10.3390/s26123715

**Published:** 2026-06-11

**Authors:** Lin Li, Keya Yuan, Yuan Chen

**Affiliations:** 1 College of Applied Science and Technology, Beijing Union University, Beijing 100101, China; ll_yykj@buu.edu.cn; 2 College of Robotics, Beijing Union University, Beijing 100101, China; 3 Jiangsu Industrial Technology Engineering Center for Environmental and Meteorological Integrated Chip System, Nanjing University of Information Science and Technology, Nanjing 210044, China; 202412492685@nuist.edu.cn

**Keywords:** radiation shield, air temperature observation, radiation-induced temperature deviation, computational fluid dynamics, neural network correction

## Abstract

**Highlights:**

**What are the main findings?**

- The proposed correction method significantly improved the accuracy of air temperature measurements by reducing radiation-induced errors.
- The proposed system achieves high-accuracy air temperature measurements under actual atmospheric conditions.

**What are the implications of the main findings?**

- The proposed approach improves air temperature observation accuracy under strong solar radiation and weak ventilation conditions.
- Integrating radiation shielding and data-driven correction provides a scalable strategy for reliable meteorological measurements.

**Abstract:**

Accurate air temperature observation requires minimizing solar radiation-induced deviations, which are strongly influenced by radiation shield performance. However, conventional shields often produce significant errors under strong solar radiation or weak ventilation. In this study, an air temperature observation system integrating a radiation shield and a backpropagation (BP) neural network-based correction method is proposed. Computational fluid dynamics (CFD) simulations were conducted to quantify radiation-induced temperature deviations under representative meteorological conditions, and the simulated dataset was used to train and test the neural network model. Initial field comparison experiments were performed using a 076B forced-ventilation system as a reference, where measured differences were treated as experimental deviations and model outputs as predicted deviations. The results show that, before correction, the proposed system exhibited a maximum deviation of 1.05 °C and a mean deviation of 0.26 °C, while the root mean square error and mean absolute error between experimental and predicted deviations were 0.30 °C and 0.23 °C, respectively. The correction significantly reduced temperature deviations, demonstrating the effectiveness of the proposed system in improving measurement accuracy.

## 1. Introduction

Surface air temperature is one of the most fundamental variables in meteorological observation, supporting a wide range of applications such as weather forecasting, climate monitoring, agricultural meteorology, environmental assessment, and urban heat island research. Its measurement accuracy directly affects the quality of meteorological services and the reliability of scientific studies [1]. With the intensification of global warming and the increasing frequency of extreme weather events, higher demands are being placed on the temporal resolution and spatial representativeness of surface air temperature observations [2]. Automatic weather stations (AWSs), owing to their efficiency, continuity, and fully automated operation, have largely replaced manual observations and are now widely deployed worldwide [3]. Despite these advantages, AWS-based temperature measurements remain susceptible to non-meteorological environmental factors such as solar radiation, wind speed variability, ventilation efficiency, surface reflectivity, and sensor thermal inertia, all of which may introduce significant radiation-induced temperature deviations [4,5].

Previous studies have shown that under clear-sky and low-wind conditions, direct solar radiation can cause temperature overestimations of approximately 4 °C [6]. Conventional naturally ventilated temperature observation systems often perform poorly under strong solar radiation and weak wind conditions, whereas forced-ventilation temperature observation systems, though more effective, are constrained by high power consumption and reliance on stable power supply [7]. Therefore, the systematic identification, quantification, and correction of temperature deviations in AWS measurements are essential for improving dataset accuracy and improving inter-station comparability, while also supporting the standardization and intelligent development of next-generation observation systems. In this context, the integration of artificial intelligence and big data analytics into radiation-induced temperature deviation correction has emerged as a promising strategy to overcome the limitations of traditional approaches and enhance data quality under complex environmental conditions [8,9].

Radiation shielding has long been a central topic in efforts to improve temperature measurement accuracy. Existing solutions include Stevenson screens, naturally ventilated shields, and aspirated (forced-ventilation) shields, each adapted to specific climatic and operational conditions but all presenting inherent limitations. Stevenson screens, historically widely used, provide stable shielding but exhibit high thermal inertia and slow response under low-wind or strong-radiation conditions [10]. With the widespread adoption of AWSs, naturally ventilated shields have become the mainstream solution due to their compact structure and ease of deployment. However, numerous studies have demonstrated that their performance deteriorates significantly under weak-wind and strong-radiation conditions. In particular, under conditions of low wind speed and high solar radiation, radiation-induced temperature deviations can exceed 3 °C and show strong sensitivity to solar elevation angle and surface albedo. Reflected solar radiation—especially from high-albedo surfaces such as snow—can further amplify sensor heating, and even forced-ventilation shields are not fully immune to this effect [11]. Forced-ventilation shields address these issues by enhancing airflow and heat exchange through active aspiration, thereby achieving high measurement accuracy across a wide range of environmental conditions [7,12]. Nonetheless, their high energy consumption, structural complexity, and dependence on continuous power supply restrict their application in remote or unattended stations.

To overcome these limitations, recent research has explored optimizing shield geometry and materials and combining radiation heat transfer models with machine learning-based post-processing [13,14]. Although these efforts have improved performance, achieving a balance among structural simplicity, low power consumption, and high measurement accuracy remains challenging—particularly in extreme environments such as high-altitude plateaus, polar regions, and urban heat islands. Radiation-induced temperature deviations primarily result from solar shortwave radiation and ground-emitted long-wave radiation, especially in naturally ventilated or low-wind conditions. Existing correction approaches can be broadly categorized into empirical regression, physical modeling, and machine learning-based methods. Empirical models, such as those proposed by Hubbard et al., are computationally efficient but have limited generalizability across environmental conditions. Physical models based on heat transfer processes offer greater interpretability but require accurate boundary conditions and are difficult to implement operationally in real time [15]. More recently, machine learning techniques have shown considerable promise, with Yang et al. employing neural networks to enhance temperature deviation correction across a wide range of environmental conditions [16]. Overall, correction methodologies are transitioning from traditional empirical and physical models toward hybrid, data-driven approaches, yet challenges remain regarding real-time applicability, environmental adaptability, and standardization.

To address these challenges, this study develops an air temperature observation system that integrates a newly designed naturally ventilated radiation shield with a backpropagation (BP) neural network-based correction model. Firstly, computational fluid dynamics (CFD) is employed to analyze the thermal and airflow characteristics of the proposed system and to quantify the radiation-induced temperature deviations under representative meteorological conditions. Then, a BP neural network correction model is constructed using the CFD-generated dataset to achieve accurate compensation of these deviations. Finally, field comparison experiments are conducted against the 076B forced-ventilation temperature observation system to validate the performance of the proposed system and the temperature deviation correction mode.

## 2. Three-Dimensional Physical Model Construction and Multiphysics Simulation of the System

### 2.1. Three-Dimensional Physical Model Construction

The radiation shield proposed in this study consists of a central hemispherical top with a diameter of 30 mm and a height of 10 mm. The upper edge slopes downward at an angle of 100°, resulting in an edge height of 10 mm, while the lower surface is inclined at 135° with an edge height of 15 mm. The central flat circular section has a radius of 20 mm, and the minimum spacing between the upper and lower surfaces is 40 mm. The shield thickness is 1 mm.

A high-precision Pt100 platinum resistance thermometer was installed at the geometric center of the shield to measure air temperature. To simulate the surrounding flow and thermal environment, a cubic computational domain of 1500 mm × 1500 mm × 1500 mm was established, providing sufficient space for the development of airflow and heat transfer around the sensor. In addition, two aluminum plates, each with a diameter of 200 mm and a thickness of 1 mm, were positioned above and below the shield with a vertical separation of 200 mm.

**Figure 1:**
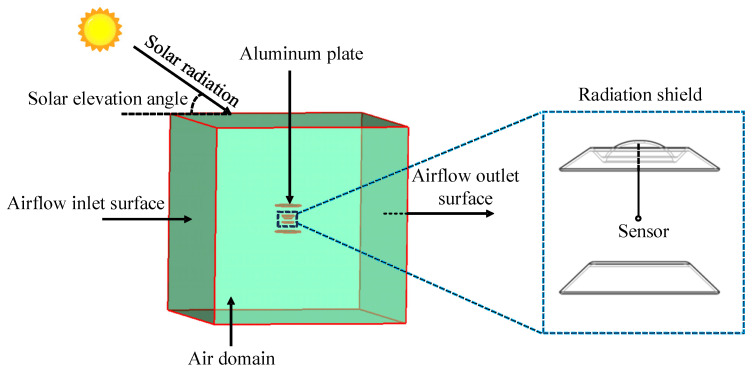
Three-dimensional physical model of the temperature observation system and its surrounding air domain.

The geometric dimensions of the radiation shield were selected based on the structural characteristics of existing radiosonde temperature sensor shields and practical installation requirements. Rather than being derived from a dedicated geometric optimization study, these dimensions were chosen to achieve a reasonable balance between solar radiation shielding effectiveness, airflow ventilation around the sensing element, and mechanical feasibility. The resulting three-dimensional physical model of the radiation shield is shown in Figure 1.

A mesh-independence study was performed using three mesh resolutions: a coarse mesh containing 1,430,867 elements, a medium mesh containing 2,178,566 elements, and a fine mesh containing 3,718,006 elements. All simulations were conducted under identical representative operating conditions, including direct solar radiation of 1000 W/m^2^, diffuse solar radiation of 200 W/m^2^, ground-emitted long-wave radiation of 300 W/m^2^, wind speed of 0.5 m/s, surface reflectivity of 0.2, and a solar elevation angle of 45°.

The predicted radiation-induced temperature deviations at the sensor location were 0.195 °C, 0.193 °C, and 0.191 °C for the coarse, medium, and fine meshes, respectively. The difference between the medium and fine meshes was only 0.002 °C (approximately 1.0%), demonstrating that further mesh refinement had a negligible effect on the simulation results. These results indicate that the predicted temperature deviation had effectively converged with respect to mesh resolution.

### 2.2. Multiphysics Simulation

**Figure 2:**
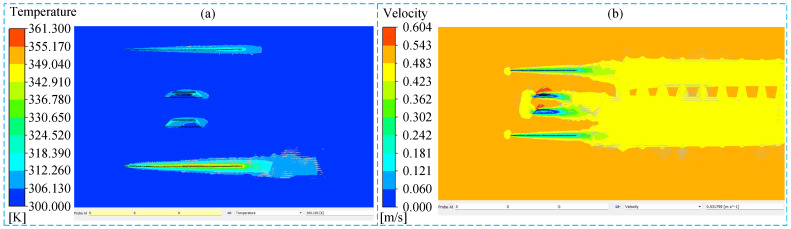
(**a**) Temperature field distribution of the temperature observation system. (**b**) Velocity field distribution of the system.

The resulting mesh was imported into FLUENT for multiphysics simulations. Subsequently, the physical models and numerical methods were specified. The *k-ε* turbulence model was employed to capture the airflow characteristics, while the SIMPLE algorithm was used for pressure–velocity coupling. Solar radiation was simulated using a solar ray-tracing model to account for direct and diffuse radiation effects on the system and surrounding surfaces [17]. In this study, the external radiative input is decomposed into four components: direct solar shortwave radiation, diffuse sky shortwave radiation, ground-reflected shortwave radiation, and long-wave thermal radiation from the sky and ground. Direct solar radiation is applied using a ray-tracing approach, with the local heat flux determined by the angle between the surface normal and the solar incidence direction. Diffuse sky shortwave radiation is assumed to be isotropic over the upper hemisphere. Ground-reflected shortwave radiation is modeled based on surface albedo and applied as diffuse reflection to the irradiated surfaces. For long-wave radiation, the radiative transfer process is solved using the discrete ordinates (DO) model, with the sky and ground treated as equivalent gray-body boundaries. The boundary conditions were defined as follows: the inlet was specified as a velocity inlet, and the outlet as a pressure outlet. The aluminum plates were assigned an outer-surface reflectivity of 95% and an inner-surface absorptivity of 90%, whereas the epoxy-resin radiation shield was assigned an absorptivity of 13%. Finally, the environmental parameters were set as follows: solar radiation intensity *P*_1_ = 1000 W/m^2^, ambient wind velocity *V* = 0.5 m/s, underlying surface reflectivity *f* = 0.2, air density *ρ* = 1.225 kg/m^3^, diffuse solar radiation intensity *P*_2_ = 200 W/m^2^, ground-emitted long-wave radiation intensity *P*_3_ = 300 W/m^2^, and solar elevation angle *E* = 45°. The resulting temperature and velocity fields obtained from the simulations are presented in Figure 2.

Under the prescribed simulation conditions—direct solar radiation intensity of 1000 W/m^2^, diffuse solar radiation intensity of 200 W/m^2^, ground-emitted long-wave radiation intensity of 300 W/m^2^, ambient wind velocity of 0.5 m/s, underlying surface reflectivity of 0.2, solar elevation angle of 45°, and air density of 1.225 kg/m^3^—the velocity field analysis shows that the local airflow velocity near the sensor probe reaches approximately 0.53 m/s when the probe is positioned at the geometric center of the model (0, 0, 0) (Figure 2b). The corresponding temperature field indicates a radiation-induced temperature deviation of 0.19 K (Figure 2a). Given the limited airflow within the shielded domain, the thermal response is jointly governed by radiative heating and weak convective cooling. The resulting deviation remains small, demonstrating that the system effectively suppresses radiative heating of the probe and ensures that the measurement accuracy is adequate for practical meteorological applications.

Although the temperature legend extends to 361 K (88 °C), such high temperatures occur only in localized regions directly exposed to intense solar radiation and correspond to the sensor housing surface rather than the ambient air temperature. The ambient airflow temperature remains close to the prescribed inlet temperature of 300 K.

### 2.3. Influence of Environmental Variables on Radiation-Induced Temperature Deviation

**Figure 3:**
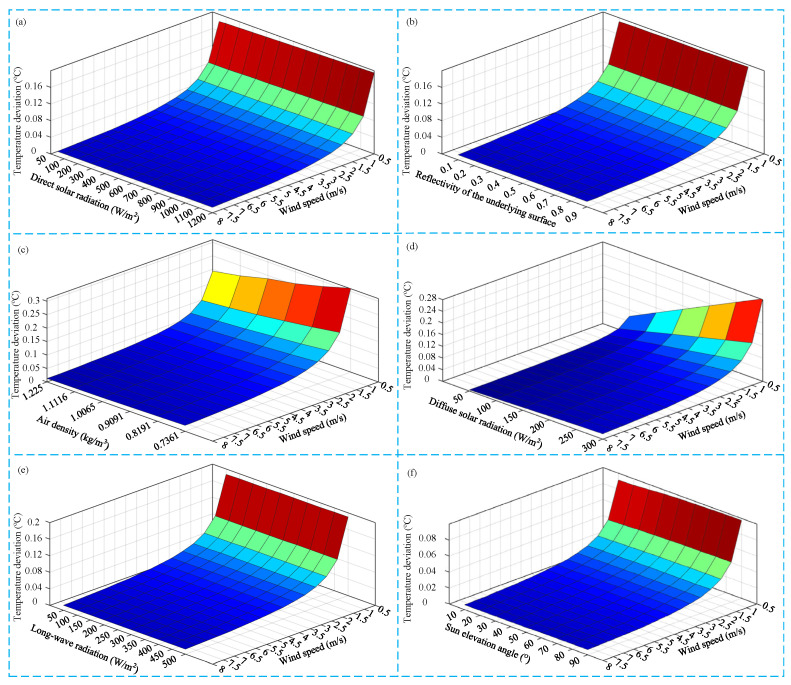
Computed radiation-induced temperature deviations under varying environmental conditions: (**a**) direct solar radiation; (**b**) underlying surface reflectivity; (**c**) air density; (**d**) diffuse solar radiation; (**e**) ground-emitted long-wave radiation; and (**f**) solar elevation angle.

In the CFD model, the environmental parameters were systematically varied within the following ranges: direct solar radiation intensity *P*_1_ = 50–1200 W/m^2^, diffuse solar radiation intensity *P*_2_ = 50–300 W/m^2^, ground-emitted long-wave radiation intensity *P*_3_ = 50–500 W/m^2^, ambient wind speed *V* = 0.5–8 m/s, underlying surface reflectivity *f* = 0.1–0.9, air density *ρ* = 0.7361–1.225 kg/m^3^, and solar elevation angle *E* = 10–90°. By systematically perturbing these parameters across their respective ranges, a total of 472 simulation cases were generated, each yielding a radiation-induced temperature deviation. The distribution of these samples is presented in Figure 3, which serves as a comprehensive dataset for subsequent neural network-based temperature deviation correction.

The outer surface of the aluminum plate has a reflectivity of approximately 0.95, while the inner surface is coated with a black layer with an absorptivity of about 0.9. This configuration effectively reduces the influence of direct solar radiation, reflected solar radiation from ground, ground-emitted long-wave radiation and solar elevation angle on the sensor probe. Under the specific CFD conditions considered in this study (direct solar radiation of 1000 W m^−2^, diffuse solar radiation of 200 W m^−2^, surface reflectivity of 0.2, wind speed of 0.5 m s^−1^, etc.), diffuse radiation exhibited a relatively strong influence on the radiation-induced temperature deviation. Specifically, when diffuse radiation increased from 50 W m^−2^ to 300 W m^−2^, the predicted temperature deviation increased from 0.058 °C to 0.283 °C, whereas the effects of several other variables were comparatively smaller over their respective ranges (Figure 3d).

In contrast, the effects of direct solar radiation, reflected solar radiation from ground, and ground-emitted long-wave radiation are comparatively small. At a wind speed of 0.5 m/s, increasing the direct solar radiation intensity from 50 W/m^2^ to 1200 W/m^2^ results in only a slight increase in temperature deviation from 0.183 °C to 0.195 °C (Figure 3a). Similarly, increasing the underlying surface reflectivity from 0.1 to 0.9 causes only a marginal change in temperature deviation from 0.192 °C to 0.199 °C (Figure 3b). Enhancing the ground-emitted long-wave radiation intensity from 50 W/m^2^ to 500 W/m^2^ leads to a minor increase in temperature deviation from 0.191 °C to 0.195 °C (Figure 3e).

Altitude has a more pronounced effect on radiation-induced temperature errors due to the reduction in air density at higher elevations, which diminishes convective heat dissipation. At a wind speed of 0.5 m/s, the radiation-induced temperature deviation increases from 0.193 °C at sea level (air density 1.225 kg/m^3^) to 0.312 °C at 5 km altitude (air density 0.7361 kg/m^3^) (Figure 3c). Regarding the solar elevation angle, when the angle increases from 10° to 90° in 10° increments under a wind speed of 0.5 m/s, the corresponding temperature deviations are 0.195 °C, 0.195 °C, 0.194 °C, 0.193 °C, 0.192 °C, 0.19 °C, 0.187 °C, 0.184 °C, and 0.183 °C, respectively (Figure 3f). Wind speed is the dominant factor governing convective cooling. As wind speed increases, the radiation-induced temperature deviation decreases substantially. When wind speed exceeds 1.5 m/s, the temperature deviation remains below 0.1 °C, highlighting the decisive role of airflow in mitigating radiation-induced temperature measurement deviations (Figure 3).

## 3. Design of the Temperature Deviation Correction Algorithm

Since CFD simulations can only capture a limited number of radiation-induced temperature deviation and are computationally demanding, a BP neural network was employed to further process the simulation data and provide radiation-induced temperature deviation estimates under generalized environmental conditions [18]. The resulting correction model establishes the quantitative relationship between the radiation-induced temperature deviation Δ*T* and the influencing variables, including direct solar radiation (*P*_1_), ambient wind speed (*V*), underlying surface reflectivity (*f*), air density (*ρ*), diffuse solar radiation (*P*_2_), ground-emitted long-wave radiation (*P*_3_), and solar elevation angle (*E*), as expressed by:

∆*T* = purelin[tansig(*P*_1_·*w_i_*_1_ + *V*·*w_i_*_2_ + *f*·*w_i_*_3_ + *ρ*·*w_i_*_4_ + *P*_2_·*w_i_*_5_ + *P*_3_·*w_i_*_6_ + *E*·*w_i_*_7_ + *θ_i_*)·*w_ki_* + *a_k_*], (1)

where the parameters *w_i_*_1_, *w_i_*_2_, *w_i_*_3_, *w_i_*_4_, *w_i_*_5_, *w_i_*_6_, *w_i_*_7_ represent the weights connecting the input layer to the hidden layer, corresponding to the seven input variables *P*_1_, *V*, *f*, *ρ*, *P*_2_, *P*_3_, and *E*. The term *θ_i_* denotes the bias of the hidden layer neurons. Similarly, *w_ki_* and *a_k_* represent the weights and bias connecting the hidden layer to the output layer, respectively.

The mathematical expressions of the tansig and purelin activation functions are given in Equations (2) and (3), respectively.

(2) $$tansig\;\left(x\right)=\frac{2}{1+\exp\left(-2x\right)}-1,$$

**Figure 4:**
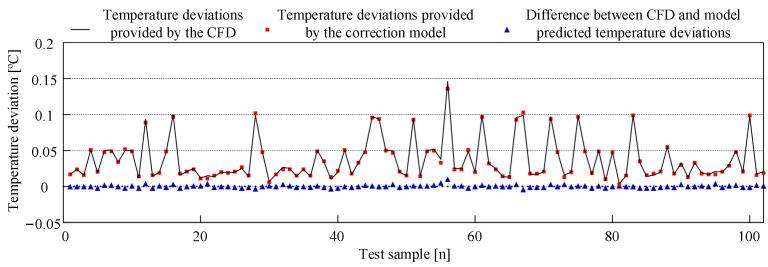
Comparison of radiation-induced temperature deviations as obtained from CFD simulations and the correction model, along with the differences between them.

(3) purelin (x)=x,

In this BP neural network model, the numbers of neurons in the input, hidden, and output layers are 7, 16, and 1, respectively. Seventy-five percent of the dataset presented in Figure 3 was randomly selected as training samples to train the model and estimate the radiation-induced temperature deviation of the proposed system, while the remaining 25% were used as test samples. The performance of the correction model was then evaluated by comparing the radiation-induced temperature deviations obtained from the CFD simulations of the test samples with those predicted by the model (Figure 4).

To quantitatively assess the predictive accuracy of the BP neural network, three widely used performance metrics were employed: root mean square error (RMSE), mean absolute error (MAE), and correlation coefficient (*r*).

The complete CFD dataset consisted of 472 samples. To assess the robustness and generalization capability of the neural network, a 10-fold cross-validation procedure was performed. The dataset was randomly divided into ten subsets; in each iteration, nine subsets were used for training and the remaining subset was used for validation. For the selected 7–16–1 network architecture, the average MAE and RMSE obtained from the ten validation runs were 0.001140 ± 0.000539 °C and 0.001784 ± 0.001209 °C, respectively. The extremely small prediction errors and the strong correlation between the neural network predictions and CFD outputs indicate that the proposed BP neural network can accurately capture the nonlinear relationship embedded in the CFD-generated dataset. These results demonstrate that the neural network provides a highly accurate and computationally efficient surrogate representation of the CFD response surface across the simulated environmental conditions. However, the practical correction capability of the method under real field conditions must be assessed separately through experimental validation.

## 4. Initial Field Comparison Experiment Research

### 4.1. Construction of the Experimental Platform

**Figure 5:**
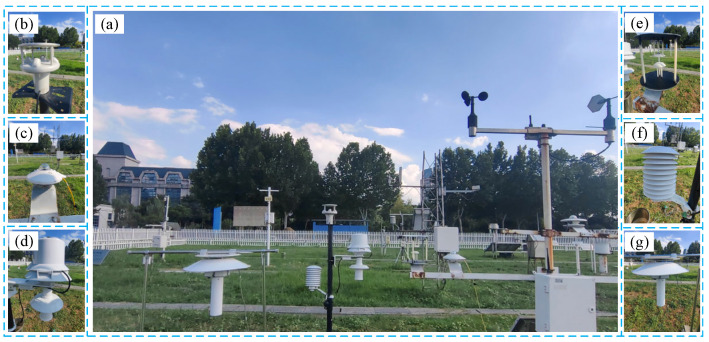
(**a**) Radiation-induced temperature deviation test platform; (**b**) ultrasonic anemometer; (**c**) CMP10 solar radiation sensor; (**d**) Model 43502 forced-ventilation temperature observation system; (**e**) proposed system; (**f**) Model 41003 naturally ventilated temperature observation system; (**g**) Model 076B forced-ventilation temperature observation system.

To evaluate the radiation-shielding performance of the proposed design and validate the simulation results, an outdoor experimental platform was established at the observation site of the Meteorological Observation Center of the China Meteorological Administration (Nanjing, China; 32.12° N, 118.42° E; altitude 22 m). The radiation-induced temperature deviation test platform is shown in Figure 5.

An aspirated 076B temperature sensor was employed as the reference instrument. According to the manufacturer’s specifications, the measurement accuracy of the sensor is ±0.03 °C. Because this error is substantially smaller than the radiation-induced temperature deviations investigated in this study, its influence on the deviation assessment can be considered negligible. For example, when the radiation-induced temperature deviation is 1.0 °C, the uncertainty of the reference sensor contributes only 3% of the reported deviation [19]. Therefore, the 076B sensor provides a reliable reference for evaluating the performance of the proposed radiation shield and for comparing experimental measurements with CFD predictions.

### 4.2. Environmental Parameters

**Figure 6:**
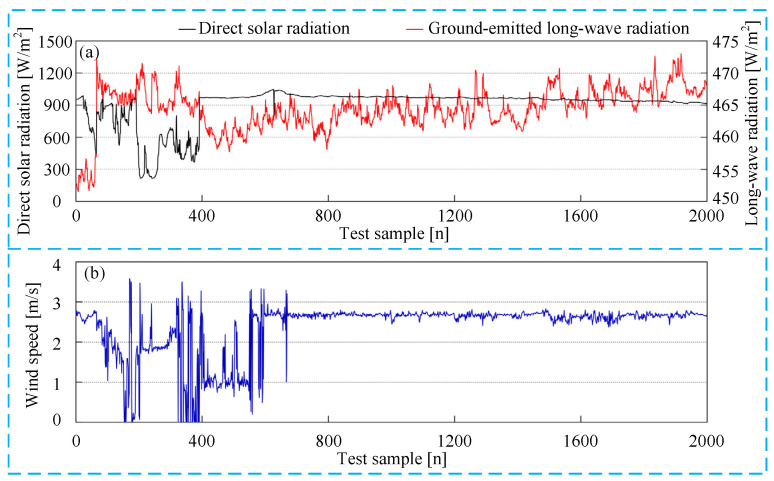
Observed environmental parameters: (**a**) direct solar radiation and ground-emitted long-wave radiation; (**b**) wind speed.

During the field experiments, global solar radiation and wind speed were measured using a CMP10 pyranometer and an ultrasonic anemometer, respectively. Since the observation site was covered by grassland, the surface reflectivity was assumed to be 0.2. In addition, because the emissivity of natural surfaces in the long-wave infrared band exhibits relatively small variations, the underlying surface was approximated as a gray body. Based on these assumptions, the ground-emitted long-wave radiation was calculated using Equation (4), where $T_{g}$ denotes the surface temperature [20]. All sensors operated at a sampling frequency of 1 Hz. To reduce the influence of short-term fluctuations and ensure consistency among different measurements, 1-min averaged values were calculated and used for subsequent analysis and model validation. The corresponding environmental parameters, including solar radiation, wind speed, and ground-emitted long-wave radiation, recorded during the experimental period are presented in Figure 6.

(4) $$I_{0}^{l}=0.95\sigma T_{g}^{4},$$

where σ represents the Stefan–Boltzmann constant.

During the field campaign, the direct solar radiation ranged from 215 to 1048 W/m^2^, the wind speed ranged from 0 to 3.58 m/s, and the ground-emitted long-wave radiation ranged from 451 to 473 W/m^2^. All of these environmental parameters fell within the ranges covered by the 472 CFD simulation samples used for neural network training. Therefore, the proposed correction model was applied primarily within the interpolation domain of the training dataset rather than under extrapolation conditions, reducing the uncertainty associated with predictions beyond the sampled parameter space.

To assess the accuracy of the CFD model, 20 experimental cases were selected, and the corresponding environmental conditions were used as boundary conditions for numerical simulations. Comparison between the measured and simulated temperatures showed a systematic deviation in the absolute temperature predictions, yielding an MAE of 2.737 °C, an RMSE of 2.745 °C, and a correlation coefficient of 0.757. Although the CFD model captured the overall variation trend of temperature, the relatively large bias in absolute temperature indicates that uncertainties remain in the representation of the complex outdoor thermal environment. Therefore, the CFD model should primarily be regarded as a tool for characterizing the relative radiation-induced temperature deviation rather than for accurately predicting absolute sensor temperatures.

It should also be noted that samples corresponding to low wind speed (<1 m/s) and strong solar radiation (≥600 W/m^2^) accounted for less than 1% of the field dataset. Consequently, the statistical support for evaluating the proposed correction method under the adverse low-wind–high-radiation conditions highlighted in the Introduction remains limited. Although the correction results exhibited an encouraging performance trend within this subset, the available sample size is insufficient to draw definitive conclusions. Therefore, the current findings should be considered preliminary. Future work will focus on conducting dedicated observation campaigns during calm summer midday periods or in regions characterized by more extreme environmental conditions, such as deserts and high-altitude areas, to obtain a larger number of low-wind–high-radiation samples and enable a more rigorous evaluation of the proposed correction model.

### 4.3. Analysis of Radiation-Induced Temperature Deviations Across Different Observation Systems

**Figure 7:**
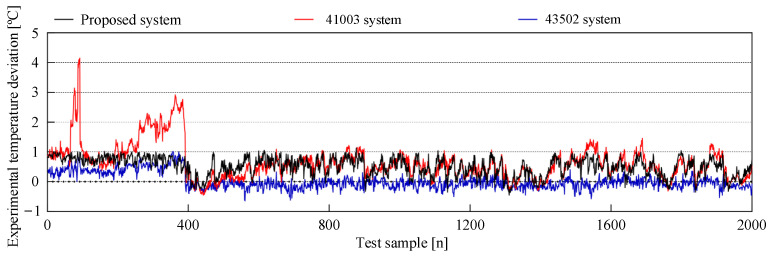
Comparison of experimental radiation-induced temperature deviations among the proposed system, the 41003 system, and the 43502 system.

The difference between the temperature measured by the proposed system and the reference temperature obtained from the 076B system represents the experimental radiation-induced temperature deviation. The experimental radiation-induced temperature deviations of the proposed system, the 41003 system, and the 43502 system are compared in Figure 7.

As shown in Figure 7, the maximum radiation-induced temperature deviations for the proposed naturally ventilated air temperature observation system, the 41003 naturally ventilated air temperature observation system, and the 43502 forced-ventilation air temperature observation system are 1.05 °C, 3.65 °C, and 0.86 °C, respectively, while the corresponding mean deviations are 0.26 °C, 0.41 °C, and 0.30 °C. The proposed system exhibits improved ventilation efficiency and enhanced radiation shielding. Consequently, its radiation-induced measurement error is reduced by approximately 37% compared with that of the 41003 system. In contrast, the proposed system does not show a significant improvement in measurement accuracy relative to the 43502 system. This is primarily because the 43502 system achieves strong ventilation performance through its fan-assisted airflow and also provides effective radiation shielding. Nevertheless, as a forced-ventilation system, the 43502 requires higher power consumption and more frequent maintenance, which represents a notable disadvantage compared with the proposed passive design.

### 4.4. Analysis of Experimental and Algorithm-Predicted Radiation-Induced Temperature Deviations of the Proposed System

**Figure 8:**
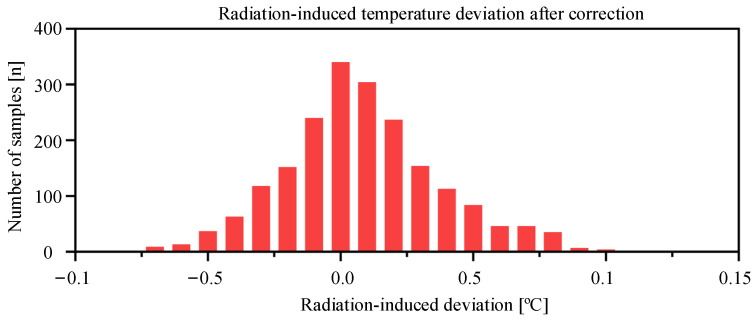
Radiation-induced temperature deviations of the proposed system after correction.

The radiation-induced temperature deviation correction algorithm trained for each experiment was applied to predict the corresponding algorithm-predicted temperature deviations. Radiation-induced temperature deviations after correction of the proposed system were the difference between the experimental and algorithm-predicted temperature deviations. A comparison of the experimental, algorithm-predicted, and corrected radiation-induced temperature deviations is presented in Figure 8.

To evaluate the practical performance of the proposed correction model, an independent field dataset was used to compare the neural-network-predicted radiation-induced temperature deviations with the experimentally observed deviations. In this validation, the measured environmental parameters were directly used as inputs to the neural network, and the predicted temperature deviations were compared with the corresponding experimental values.

The results show that the MAE and RMSE between the predicted and experimental radiation-induced temperature deviations were 0.23 °C and 0.30 °C, respectively. Considering that the neural network was trained entirely using CFD-generated samples, these results indicate a reasonable agreement between the predicted and measured deviations under real atmospheric conditions. Furthermore, after applying the correction model, the mean radiation-induced temperature deviation was reduced to 0.07 °C (Figure 8), demonstrating that the proposed method can effectively mitigate radiation-induced measurement bias. As shown in Figure 8, approximately 56% of the corrected samples exhibit residual temperature deviations within ±0.2 °C. This proportion increases to approximately 65% and 73% when the error threshold is expanded to ±0.25 °C and ±0.3 °C, respectively, indicating that most corrected samples are concentrated near zero residual error.

The discrepancy between the field-validation results and the significantly smaller errors obtained during CFD-based validation mainly arises from the complexity of real atmospheric conditions. Several factors may contribute to the residual errors, including the measurement uncertainty of the 076B aspirated reference system (approximately ±0.03 °C), uncertainties associated with the CFD model, and environmental processes that were not explicitly considered in the simulations, such as rapid cloud-induced radiation fluctuations, turbulent wind-speed variations, indirect humidity effects, and minor dust accumulation on sensor surfaces. Collectively, these factors introduce additional uncertainty into the correction process under field conditions.

To further quantify the effectiveness of the correction, the statistical characteristics of the temperature deviations before and after correction were compared. The mean bias error (MBE) decreased from 0.281 °C before correction to −0.014 °C after correction, indicating that the systematic bias was nearly eliminated. The MAE was also reduced from 0.281 °C to 0.238 °C. However, the RMSE increased slightly from 0.340 °C to 0.359 °C, while the standard deviation increased from 0.191 °C to 0.359 °C. These results suggest that the correction model primarily reduces the systematic component of the radiation-induced error but has a limited effect on the random variability of the residuals. Therefore, the principal advantage of the proposed method lies in bias correction rather than in suppressing short-term fluctuations caused by complex environmental disturbances.

## 5. Conclusions

In this study, an air temperature observation system integrating a newly designed naturally ventilated radiation shield with a BP neural network-based correction model was developed and experimentally evaluated. CFD simulations were first performed to quantify radiation-induced temperature deviations under representative meteorological conditions, and the resulting dataset was used to train the neural network. Independent field experiments were then conducted using a 076B aspirated radiation shield as the reference system.

The results indicate that, within the environmental conditions covered by the present field experiment (Nanjing grassland site, wind speeds of 0–3.58 m s^−1^, and direct solar radiation ranging from 215 to 1048 W m^−2^), the proposed correction approach effectively reduced radiation-induced measurement bias. After correction, the mean radiation-induced temperature deviation decreased to 0.07 °C. Furthermore, approximately 56%, 65%, and 73% of the corrected samples exhibited residual temperature deviations within ±0.2 °C, ±0.25 °C, and ±0.3 °C, respectively. The comparison between the predicted and experimentally observed temperature deviations yielded an MAE of 0.23 °C and an RMSE of 0.30 °C, indicating reasonable agreement between the correction model and field observations. Compared with conventional naturally ventilated radiation shields, the proposed system reduced radiation-induced measurement bias by approximately 37% while maintaining the advantages of low power consumption, simple structure, and low maintenance requirements.

The results demonstrate the feasibility of combining radiation-shield design with data-driven correction techniques to improve air-temperature measurements under outdoor conditions. However, several limitations should be acknowledged. First, the field validation was conducted at a single site and within a limited range of environmental conditions, and the number of samples corresponding to low-wind (<1 m/s) and strong-radiation (≥600 W/m^2^) conditions was relatively small. Second, a noticeable performance gap exists between the CFD-based validation and the field-validation results, reflecting the influence of environmental variability, measurement uncertainty, model simplifications, and other real-world factors that are difficult to fully reproduce in numerical simulations. Third, the conclusions regarding the attenuation characteristics of diffuse solar radiation are based on the specific CFD assumptions adopted in this study and therefore require further verification under diverse atmospheric conditions.

Future work will focus on expanding field validation to multiple sites, seasons, and climate regimes, particularly under low-wind and high-radiation conditions. Additional environmental variables, such as humidity and turbulence intensity, will be incorporated into the correction model to improve prediction accuracy. Furthermore, hybrid training strategies combining CFD-generated and field-observed datasets, together with improved radiative-transfer modeling and long-term operational testing, will be investigated to enhance the robustness, generalization capability, and practical applicability of the proposed system.

## Data Availability

All data supporting the findings of this study are included within the article. No additional datasets were generated or analyzed beyond those presented in the published paper.
